# Clinical efficacy of duloxetine in the treatment of axial symptoms after posterior cervical spine surgery: a retrospective study

**DOI:** 10.1186/s13018-023-03970-8

**Published:** 2023-07-12

**Authors:** Jinkai Liu, Xiaotian Yang, Wanli Jing, Xing Guo, Rui Wang, Jiaming Zhou, Yuan Xue

**Affiliations:** 1 Department of Orthopaedic Surgery, Tianjin Medical University General Hospital, Tianjin, China; 2 Department of Burn and Plastic Surgery, People’s Hospital of Chongqing Hechuan, Chongqing, China; 3 Department of Orthopaedics, Tianjin First Central Hospital, Tianjin, China; 4 Tianjin Key Laboratory of Spine and Spinal Cord, Tianjin Medical University General Hospital, Tianjin, China

**Keywords:** Axial symptoms, Cervical spondylotic myelopathy, Duloxetine, Posterior cervical spine surgery

## Abstract

**Purpose:**

To evaluate the efficacy of duloxetine in the treatment of patients with axial symptoms after posterior cervical spine surgery.

**Methods:**

Patients with axial symptoms after posterior cervical spine surgery treated by duloxetine or non-drug therapy from 2018 to 2021 were reviewed. Duloxetine was administered gradually, with oral administration of 30 mg in the first week and oral administration of 60 mg from the second week. Visual analogue scale (VAS), 36-Item Short-Form Health Survey questionnaire (SF-36) and EuroQol-5 Dimensions (EQ-5D) questionnaire were used to evaluate the severity of AS at baseline and 1 week, 2 weeks, 1 month, 3 months and 6 months after medication. The occurrence of adverse reactions was recorded.

**Results:**

A total of 63 eligible patients who received duloxetine therapy (*n* = 35) or non-drug therapy (*n* = 28) were included. All patients were followed up for 6 months. Significant improvements were found in VAS score compared with baseline in both groups (1.87 ± 0.81 vs 6.61 ± 1.16, 3.18 ± 0.67 vs 6.31 ± 1.40; *P* < 0.05 for all). Meanwhile, the VAS score of the duloxetine group was significantly better than that of the non-drug therapy group at 1 week, 2 weeks, 1 month, 3 months and 6 months (*P* < 0.05). Besides, according to 36-Item Short-Form Health Survey questionnaire (SF-36), the PCS score and MCS score are significantly higher than before the treatment in duloxetine group (PCS 62.82 ± 6.04 vs 44.36 ± 7.25, MCS 65.50 ± 4.53 vs 55.55 ± 6.06; *P* < 0.05 for all). And when we compared variables between the two groups, the PCS score of the duloxetine group was significantly better than that of the non-drug therapy group (*P* < 0.05), but there was no significant difference in MCS score between the two groups (*P > *0.05). What’s more, EQ-5D score had significant improvements in the duloxetine group compared with the non-drug therapy group at 1 week, 2 weeks, 1 month, 3 months and 6 months (*P* < 0.05).

**Conclusion:**

Oral duloxetine has a better short-term outcome than conventional non-drug therapy in patients with axial symptoms following posterior decompression surgery in the cervical spine.

## Background

Cervical spondylotic myelopathy (CSM) usually referred to a degenerative disease in which dorsal and/or ventral lesions compress the cervical spinal cord, resulting in a series of distinct clinical manifestations [1]. It is most common in middle-aged and elderly patients with loss of integrity of the intervertebral disk, facet joint and uncinate joint osteophytes and hypertrophy of the ligamentum flavum [2]. Its onset is usually relatively hidden, characterized by gait instability and fine motor defects, or nonspecific neck shoulder pain, some patients have a fine hand numbness and hand movement disorders, gait based on broad, ataxia, difficult to carry out such performance series gait, is a common cause of non-traumatic tetraplegia, severely reduces the patient’s quality of life [3, 4]. Although CSM has a wide range of clinical manifestations, most patients experience progressive disease progression with limited hope of self-healing [5, 6]. In this process, patients tend to delay seeing a doctor because the early symptoms are mild [7]. Therefore, surgical treatment is often recommended as the most effective means to limit the progression of symptoms [8].

No matter what type of operation is used, postoperative complications are always a topic of concern for spinal surgeons. Postoperative complications of posterior approach include wound infection, iatrogenic kyphosis, C5 nerve root palsy and axial symptoms [9–12]. Postoperative complications not only affect patients’ postoperative recovery, but also play an important role in enhanced recovery after surgery (ERAS) [13, 14].

Axial symptoms (AS), also known as axial pain or axial neck pain, are a common complication of posterior cervical surgery [12]. First reported by Hosono in 1996 [15], Kawaguchit et al. [16] concluded that patients experienced chronic neck, shoulder and back muscle spasm, pain and stiffness after cervical spine surgery, which in severe cases affected patients’ work and life. In posterior approach, the incidence is 5.2–80%. Persistent axial pain may be a major cause of postoperative dissatisfaction, even in patients with good neurological recovery [17]. At present, the cause of axial symptoms is not clear, and some studies believe that AS is related to the destruction of the posterior muscular ligament complex of cervical spine [18]. Some studies have suggested that AS is related to decreased cervical motion after surgery [19–21]. Some studies have even found that AS is related to postoperative mental state of patients [22]. Therefore, it is extremely important to find effective treatments for AS to promote ERAS in patients.

At present, one of the most popular explanations for chronic pain is central pain sensitization. Studies have shown that the imbalance of serotonin and norepinephrine system in central pain pathway plays an important role in the development of pain sensitivity [23, 24]. Duloxetine is a selective serotonin and norepinephrine reuptake inhibitor, which is effective for severe depression, generalized anxiety disorder and fibromyalgia. Studies have shown [25, 26] that duloxetine can effectively treat three different kinds of chronic pain: diabetic peripheral neuralgia, neuralgia and chronic low back pain. The research of Chappell et al. [27] shows that duloxetine can significantly relieve pain and improve function in the treatment of chronic pain caused by knee osteoarthritis. Moreover, in China, the indication of duloxetine in chronic musculoskeletal pain was approved from September 2018 [28]. However, duloxetine in the treatment of axial neck pain has not been reported.

In this study, we hypothesized that oral duloxetine has a better short-term outcome in patients with axial symptoms. Hence, the purpose of this retrospective study was to investigate the clinical efficacy of duloxetine in the treatment of axial symptoms after posterior cervical decompression.

## Methods

### Patient demographics

Patients with axial symptoms after posterior cervical decompression surgery in the Tianjin Medical University General Hospital from 2018 to 2021 were analyzed retrospectively. AS are defined as long-term neck and shoulder back muscle spasm, acid swelling pain and heavy stiffness after cervical surgery [16]. The observation time was 6 months.

Inclusion criteria were: Posterior cervical surgery was performed in our hospital, and AS occurred after operation. Exclusion criteria were: (1) rheumatic or rheumatoid arthritis or other serious systemic diseases; (2) history of mental disorders, including severe depression; (3) history of substance abuse or dependence; and (4) lack of sufficient follow-up data. Finally, a total of 63 patients were included in this study.

### Treatment

Between January 2018 and May 2019, patients with AS received non-drug therapy, including health education, functional exercise and cervical collar wearing; after May 2019, patients suffered AS were given duloxetine. Hence, patients were divided into two groups: the non-drug group (*n* = 28) and duloxetine group (*n* = 35). The baseline data of all patients were from clinical medical records, including age, gender, smoking, body mass index (BMI), length of hospital stay, appearance time of AS, duration of symptoms before medication and mode of operation. The initial VAS score, SF-36 score and EQ-5D score of all patients were also recorded. Duloxetine (Cymbalta®) 60 mg once a day, 30 mg orally in the first week and 60 mg orally from the second week. At each follow-up, patients’ data were collected.

### Post-intervention assessment

Visual analogue scale (VAS): a visual analogue scale from 0 to 10, which is used to evaluate the degree of neck pain [29, 30]. A score of 0 indicates no pain, and a score of 10 indicates the most unbearable pain. Clinical evaluation: “0–2” is “excellent,” “3–5” is “good,” “6–8” is “fair,” and “8–10” is “poor” [31–33].

SF-36 life index score (36-item short-form health survey questionnaire): It is a very popular questionnaire to evaluate health-related quality of life. It is mainly composed of 8 sections: physical function (PF), occupational-related physical factors (RP), general health status (GH), physical pain score (BP), social ability assessment (SF), occupational-related mental factors (RE), mental state assessment (VT) and mental health score (MH). Among them, PF, RP, GH and BP mainly evaluate the physical dimension of subjects, that is, physical condition (PCS), while SF, VT, re and MH focus on the mental level, that is, mental health (MCS).

EQ-5D questionnaire (EuroQol five dimensions questionnaire): It is a general health status measurement tool developed by the international research organization EuroQol group. It evaluates the health status of the population in the form of questionnaire and describes the quality of life. It mainly includes five dimensions: activity ability, self-care, daily activities, pain/discomfort, and anxiety/depression. Each dimension is divided into three levels: no difficulties, some difficulties and extreme difficulties.

### Power analysis

Based on previous studies and our pilot experiment, we assumed normal distribution and a VAS standard deviation (SD) of 1.0. With a two-sided *α* = 0.05, a sample size of 25 patients in each group gave a power of 0.8 to detect a mean difference of 0.5 in VAS.

### Statistical analysis

The intensity of axial symptoms was evaluated by VAS score, and the quality of life was evaluated by SF-36 score and EQ-5D score. VAS score, SF-36 score and EQ-5D score were recorded at baseline and 1 week, 2 weeks, 1 month, 3 months and 6 months after medication. The occurrence of adverse reactions was recorded.

All relevant data were collected and statistically evaluated by SPSS software version 22.0 (IBM Corporation, Armonk, NY, USA). The differences between the two groups were compared by independent sample t test and chi-square test. Independent sample t test was used to evaluate the clinical results of the two groups at 1 week, 2 weeks, 1 month, 3 months and 6 months. *P* value of < 0.05 was considered statistically significant.

## Results

### Baseline characteristics

A total of 63 patients (49 men and 14 women) were included in this study. The demographic data of patients are shown in Table 1. There was no significant difference in age, gender, smoking, body mass index (BMI), length of hospital stay, appearance time of AS, duration of symptoms before medication, mode of operation, initial SF-36 score and initial EQ-5D score between the two groups.

**Table 1:** Premedication data of two groups

	Duloxetine group (*n* = 35)	Non-drug therapy group (*n* = 28)	*P* value
Age (years)	66.62 ± 5.83	65.69 ± 5.26	0.511
Gender (male) (%)	28(80.0)	21(75.0)	0.635
Smoking (%)	23(65.7)	18(64.3)	0.906
BMI (kg/m^2^)	26.06 ± 1.41	25.67 ± 2.32	0.577
Length of stay (days)	15.97 ± 4.80	15.54 ± 3.46	0.691
Appearance time of AS (days)	4.39 ± 2.61	4.51 ± 0.91	0.792
Duration of symptoms before medication(days)	15.68 ± 3.93	15.25 ± 4.26	0.667
VAS scores	6.61 ± 1.16	6.31 ± 1.40	0.371
SF-36 scores			
PCS	44.36 ± 7.25	42.42 ± 6.31	0.269
MCS	55.55 ± 6.06	54.00 ± 4.86	0.446
EQ-5D	0.518 ± 0.186	0.497 ± 0.176	0.757
Operative style			0.908
Laminectomy	10	8	
Laminoplasty	21	17	
Hybrid surgery	4	3	

*BMI* body mass index, *VAS* visual analog scale, *SF-36* 36-Item short-form health survey questionnaire, *EQ-5D* EuroQol-5 Dimensions questionnaire

### Treatment effect on study parameters

**Fig. 1:**
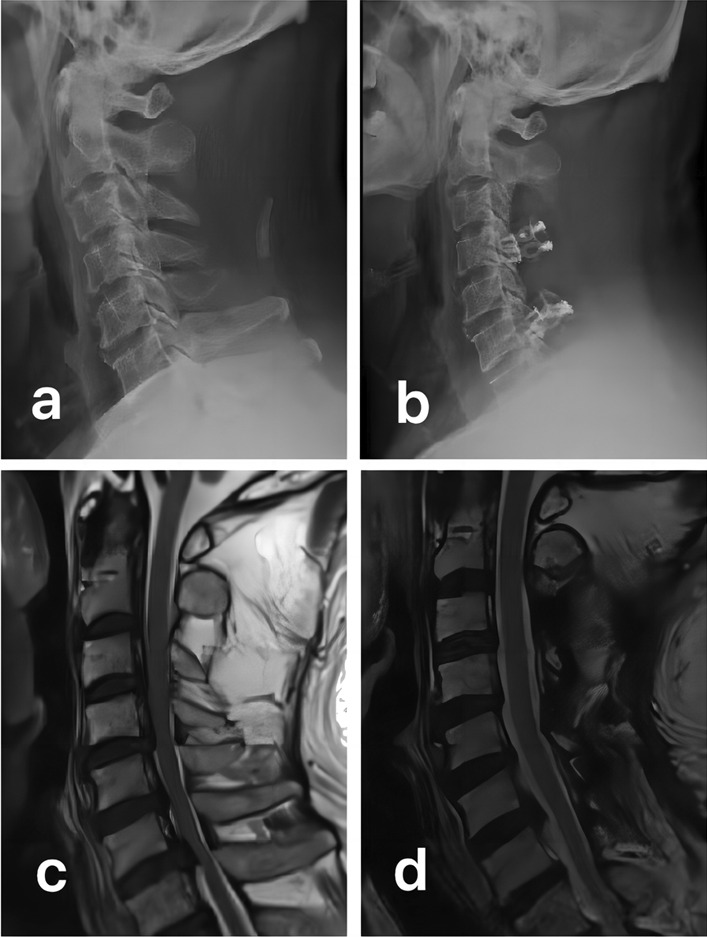
A 57-year-old patient who underwent posterior cervical decompression: multi segmental compression was seen before operation (**a**, **c**); after operation, sufficient decompression was observed, and cervical range of motion was satisfactory. Axial symptoms occurred 7 days after surgery. Under the treatment of duloxetine, the patients’ neck pain was relieved and function was improved (**b**, **d**)

As shown in Table 2, symptoms of the neck pain decreased over time in both groups (1.87 ± 0.81 vs 6.61 ± 1.16, 3.18 ± 0.67 vs 6.31 ± 1.40; *P* < 0.05 for all). And the VAS score in duloxetine group was significantly better than that of the non-drug therapy group at 1 week, 2 weeks, 1 month, 3 months and 6 months (*P* < 0.05). Meanwhile, according to Table 3, the PCS score and MCS score are significantly higher than before the treatment in duloxetine group (PCS 62.82 ± 6.04 vs 44.36 ± 7.25, MCS 65.50 ± 4.53 vs 55.55 ± 6.06; *P* < 0.05 for all). And when we compared variables between the two groups, duloxetine group showed a significant difference in PCS score (*P* < 0.05). But there was no significant difference in MCS score between the two groups (*P* > 0.05). What’s more, as shown in Table 4, between group differences in change of the outcome demonstrated that duloxetine group had significant improvements in EQ-5D score compared with the non-drug therapy group (*P* < 0.05). In addition, we collected and reviewed all of the imaging data. One postoperative patient with axial symptoms treated using duloxetine is illustrated in Fig. 1.

**Table 2:** VAS score of two groups

	Duloxetine group (*n* = 35)	Non-drug therapy group (*n* = 28)	*P* value
Baseline	6.61 ± 1.16	6.31 ± 1.40	0.371
1 week	3.36 ± 0.61*	5.11 ± 0.82*	< 0.001
2 weeks	3.23 ± 0.76*	4.55 ± 0.94*	< 0.001
1 month	2.42 ± 0.74*	3.89 ± 0.75*	< 0.001
3 months	2.09 ± 0.77*	3.64 ± 0.94*	< 0.001
6 months	1.87 ± 0.81*	3.18 ± 0.67*	< 0.001

*VAS* visual analog scale

Compared with the baseline values, *P < 0.05

**Table 3:** SF-36 score of two groups

	Duloxetine group (*n* = 35)	Non-drug therapy group (*n* = 28)	*P* value
*PCS scores*			
Baseline	44.36 ± 7.25	42.42 ± 6.31	0.269
1 week	55.56 ± 8.00*	46.23 ± 7.53*	< 0.001
2 weeks	57.35 ± 7.42*	49.39 ± 6.42*	< 0.001
1 month	60.41 ± 6.80*	50.02 ± 6.93*	< 0.001
3 months	61.63 ± 6.11*	51.00 ± 7.00*	< 0.001
6 months	62.82 ± 6.04*	51.42 ± 6.80*	< 0.001
*MCS scores*			
Baseline	55.55 ± 6.06	54.00 ± 4.86	0.446
1 week	62.87 ± 4.97*	61.81 ± 4.44*	0.547
2 weeks	64.02 ± 4.78*	62.86 ± 4.53*	0.505
1 month	65.01 ± 4.69*	63.40 ± 4.33*	0.335
3 months	65.08 ± 4.69*	64.05 ± 4.43*	0.540
6 months	65.50 ± 4.53*	64.36 ± 4.36*	0.491

*SF-36* 36-Item Short-Form Health Survey questionnaire

Compared with the baseline values, *P < 0.05

**Table 4:** EQ-5D health status score of patients in the two groups

	Duloxetine group (*n* = 35)	Non-drug therapy group (*n* = 28)	*P* value
Baseline	0.518 ± 0.186	0.497 ± 0.176	0.757
1 week	0.656 ± 0.135	0.561 ± 0.164	0.031
2 weeks	0.698 ± 0.130	0.573 ± 0.165	0.029
1 month	0.711 ± 0.129	0.577 ± 0.166	0.020
3 months	0.738 ± 0.111	0.578 ± 0.133	< 0.001
6 months	0.742 ± 0.107	0.583 ± 0.106	< 0.001

*EQ-5D* EuroQol-5 dimensions questionnaire

### Complications

The side effects of duloxetine in 35 patients in duloxetine group were statistically analyzed. Adverse effects in the duloxetine group were nausea (2; 5.71%), dizziness (1; 2.86%), fatigue (1; 2.86%) and abdominal distension (1; 2.86%).

## Discussion

In this retrospective study, we observed that oral duloxetine has better short-term efficacy in patients with axial symptoms after posterior cervical decompression than conventional conservative treatment. At 1 week, 2 weeks, 1 month, 3 months and 6 months after medication, the VAS scores and EQ-5D scores of the duloxetine group were significantly better than that of the non-drug therapy group. Meanwhile, the PCS score and MCS score are significantly higher than before the treatment in duloxetine group. This drug treatment can help reduce the postoperative neck pain and improve the quality of life of patients.

### The pathogenesis of axial symptoms

At present, the pathogenesis of AS is not clear, which is mainly related to the following reasons. One is destruction of posterior cervical muscle ligament complex. The complex formed by the posterior cervical muscles and ligaments is considered to be the key to maintaining the static stability of the cervical spine [18], which together with the cervical spine itself maintains the stability of the cervical spine. Among them, the most important is the cervical spinous muscle and its stop on C2. Traditional posterior cervical decompression surgery usually destroys this structure, resulting in cervical hemispinous muscle atrophy and the occurrence of AS. The range of motion of cervical spine decreased after operation is another reason. Orthopedic doctors usually ask patients to wear cervical collar after operation to maintain cervical stability [34]. However, prolonged neck support wearing can also lead to the occurrence of AS [19–21].

### The mechanism of duloxetine in AS

Duloxetine is a serotonin–norepinephrine reuptake inhibitor (SNRI) for the treatment of generalized anxiety disorder, severe depressive disorder and chronic pain. There is evidence that the stress system and its interaction with the nervous system and immune system play an important role in the development of persistent and chronic musculoskeletal pain [35, 36], and serotonin and norepinephrine are the most important neurotransmitters in this regard. AS is different from postoperative pain. It could last many years and cause great inconvenience to patients. In an average 14-year follow-up study, researchers found that 33% of the patients complained of AS after surgery and 28% of patients complained of AS at the final follow-up [37]. In our study, significant improvement of VAS score, PCS scores in SF-36 and EQ-5D scores was found in duloxetine group at each follow-up point, especially in 6-month follow-up, indicating that duloxetine is an ideal treatment candidate given the characteristics of AS.

### Adverse effect of duloxetine

Duloxetine has a very low anticholinergic side effect profile, adverse effects of the cardiovascular, gastrointestinal, central nervous system, such as headaches and drowsiness, and fatigue [38, 39]. The most frequent adverse reactions are sexual dysfunction, nausea, headache, dry mouth, somnolence and dizziness. Most treatment emergent adverse events and discontinuations due to adverse events occur within the first weeks of duloxetine therapy and tend to decrease over time [40]. According to previous studies, duloxetine 60 mg/day is generally safe, well tolerated and effective in reducing pain [28, 41]. In this study, although there were adverse events in the duloxetine group, they were mostly mild to moderate, and in addition, most of these adverse events occurred early in treatment and were gradually reduced.

### Evaluation of axial symptoms

At present, there is no unified standard for the evaluation of the severity of AS. In a systematic review by Duetzmann [42], it was found that less than 30% of the reports on laminoplasty from 2003 to 2013 used visual analogue scale (VAS) or other indicators to quantify AS. It is far from enough to evaluate AS only by pain intensity, because it cannot fully reflect the symptoms and severity of AS, nor can it evaluate the surgical satisfaction and quality of life of patients. Therefore, some researchers used other scales to evaluate AS. Kimura [17] paid more attention to the impact of AS on patients’ quality of life. Through SF-36 scale and EQ-5D score, he found that AS seriously affected patients’ life treatment and seriously reduced patients’ satisfaction with the treatment process. These are not evaluated by the traditional VAS score.

This experiment adopts a method similar to Kimura. Based on VAS score, SF-36 scale and EQ-5D score are introduced to evaluate patients’ operation satisfaction and quality of life. From the test results, it is not difficult to find that compared with the control group, duloxetine group patients not only have significant improvement in VAS score, but also show significant improvement in quality of life on SF-36 scale and EQ-5D scale. Duloxetine had a significant effect on AS after posterior cervical decompression, and the relief of pain also improved the quality of life and surgical satisfaction, which cannot be ignored in the process of recovery after posterior cervical surgery.

### Limitations

There are still some deficiencies in this study: First, the sample size is small. As is a common complication after posterior cervical decompression, although the incidence of AS has decreased with the improvement of surgical methods and reasonable postoperative functional exercise, the representativeness of the sample size of patients included in this study is still insufficient, which may lead to the error of the results. Second, at this stage, there is no recognized standard for the evaluation of AS. The evaluation standard adopted in this paper is presented in the form of scale, which is highly subjective. It may cause errors due to the subjects’ insufficient cognitive level or misunderstanding of the questionnaire, or the researchers’ subjective impression. In the next step, multicenter randomized control trial with long-term follow-up is needed. At the same time, various data such as imaging and laboratory can be considered to comprehensively evaluate the efficacy of duloxetine.

## Conclusion

Through this retrospective study, we found that oral duloxetine has better short-term efficacy in patients with axial symptoms after posterior cervical decompression than conventional conservative treatment.

## Data Availability

Please contact author for data requests.
